# Practical Observations on Mercurial Fumigation

**Published:** 1825-07

**Authors:** A. Gibson

**Affiliations:** Staff Surgeon.


ART. III.—*Practical Observations on Mercurial Fumigation.* By A. GIBSON, M.D. Staff Surgeon. (Communicated through Dr. JAMES JOHNSON.)

"Femur tumentibus

Exile suris additum."

OF all the duties of the military surgeon, the most constant and unsatisfactory is that which calls upon him to administer to the chronic forms of disease, usually classed in our medical Returns under the specious term "Anomalous." It is the obstinacy of these, and their perplexing variety, which, no doubt, so long ago conferred the epithet opprobrium on many of them; for, after having withstood the most accepted judicious medical treatment, and been unprofitably subjected to the not-minor benefits, in many instances, of long ease and nursing, they have usually at last been abandoned, to be ranked among the incurables, and their victims exiled beyond the pale of science.

But it has, doubtless, been observed, by every medical officer of a certain military experience in India, how frequently those men again offer themselves to the public service, full of energy and activity, and free from every physical disqualification, amply competent (as they afterwards prove) to its most arduous duties. This observation was rendered particularly remarkable last year, whilst in Rhandiesh, by the return of a Sepoy, after a short leave of absence, supple and active, although emaciated, who had left the hospital crippled and nearly helpless. The fact was too striking not to make an impression, and academic honours blushed for a moment to be outdone by some illiterate Paracelsus of a Mahratta hamlet. It was very evident, from the state of the gums and mouth in this case, that mercury had been used in some form, which had acted upon them with more severity than, in similar cases, is thought by British practitioners to be required, and that the debility at the same time was by no means such as would have followed any course of mercury prescribed, *secundum artem*, to produce equal effects. But the Sepoy was too ignorant, or his *hukkheem* too cunning; for I could not ascertain that mercury had actually been given, but only that he had been exposed to fire and smoke, and drank some medicated decoction, during which his mouth had become very sore, and he himself rapidly convalescent.

It was on being foiled in the treatment of some of the following cases, that I was led to consult the judgment of Dr. Milne, the superintending surgeon of the division, on the probable good effects to be derived from mercurial fumigation, who, ever unwearied in his zeal for the promotion of medical improvement, procured for me a skilled native, to whose method alone I entrusted my earlier cases; which, with others, equally fortunate, in my own immediate charge, form the subject of this communication.

### *Cases successfully treated by Mercurial Fumigation.*

Koondallee Sackpal, Sepoy, admitted into hospital, with rheumatism, on the 24th of June. Pain greatest in the right elbow-joint, which was considerably swollen and stiff. All the usual means recommended in such instances failing, mercurial fumigations were tried the beginning of

August, which being administered fourteen times, or morning and evening for seven days, he was returned for duty on the 15th of that month; being detained in hospital the last few days, till salivation ceased, and the mouth became well.

Balnac Darnac, admitted on the 28th of June, with rheumatism. Pains general, but greatest in the ankle-joints, which were swollen and rigid; the swelling and stiffness extending over the foot to the toe. The usual remedies failing, fumigations were begun the beginning of August, applied fourteen times; and on the 11th of July he was dismissed.

Doon Sing, Sepoy, admitted into hospital, with syphilis, on the 17th of February. A phagedenic chancre, of a most obstinate and threatening nature, was the most difficult primary symptom to treat. The usual mercurial course was followed by general pains, and all the untoward symptoms denominated "anomalous." Unsuccessful trials were made of the alteratives considered the most active,—such as oxy. mur. hydr., pil. hydr., sarsaparilla, ipecacuanha, alone and in many varied combinations with opium; blisters, liniments, cupping, frictions, and bandaging, were long and ineffectually used. He was bedrid, and his general health greatly impaired, from suffering and want of rest, when mercurial fumigations were resorted to. He is, (August 21st,) after fourteen applications, walking actively about the hospital, complaining only of a sore mouth, and very slight stiffness of the ankle-joints; which, considering the dampness of the season, is not to be wondered at.

October 27th.—Perfectly well.

Goordat Sing, Sepoy, admitted, with venereal, on the 28th of February. The early history of this patient is very similar to that of Doon Sing. The secondary character which his disease assumed was so much the more formidable, however, from swellings that from time to time appeared on different parts of his body. The principles of the treatment were the same. The following were the prominent symptoms and appearances in the beginning of August, when mercurial fumigations were used:—General health greatly impaired; bowels irregular; appetite bad; habit emaciated; occasional nocturnal attacks of fever, with intense thirst; constant lassitude and listlessness; considerable enlargement of the os calcis; swelling of the digital extremity of the middle metacarpal bone, and a bump on the spine of the scapula: these enlargements acutely sensible to the touch. As the system became affected, after a few fumigations, all external marks of disease began to vanish; the pains, from the first, became blunted; and, after seven days use of the remedy, he had no complaints. At present (August 21st,) he is active and supple, and, but for the inclemency of the season, would be on the convalescent list.

October 27th.—Stout and well.

Shaik Moriddeen, Sepoy, admitted, with rheumatism, on the 9th of May. Painful swelling and stiffness of the joints of the lower extremities; for which the usual active and palliative means were vainly used. Commenced fumigations the beginning of August, which were employed, as in the other cases, fourteen times. Was severely salivated. Dismissed, cured.

Rodrigues— was admitted on the 1st of August, with nocturnal pains, and a scabby eruption of the legs and arms. Was lately under mercury for a virulent chancre, for the usual time. After seven days' fumigation, the pains subsided, the eruption disappeared, and he was dismissed.

N.B.—September 30th. This patient, a poor man, left the hospital in damp, rainy weather, whilst under mercury, and has since sought relief for pains, most probably to be thus accounted for; and, having been again successfully fumigated, is now free from complaint.

The following cases formed the subject of a second communication to the superintending surgeon.

August.—A female, æt. seventeen. Pains and swelling of the lower extremities, (the former, worst during the night;) general pains and stiffness of the joints; great difficulty in rising, when once seated; emaciated; colour sickly; appetite bad. Has been ailing above a year. Was fumigated fourteen times; mouth became only tender; salivation very trifling. Cured.

*Remarks.*—Although the history of this female was imperfect, the symptoms afford grounds for suspecting a venereal taint, but she persists in never (to her knowledge) having been diseased. But, venereal or rheumatic, the case is valuable, the cure being complete.

A commissariat servant, was supposed to be cured of chancres about two months ago, after the usual course of mercury, in the form of pill and friction. For a week past, has been troubled with a thickening and dryness of the cuticle, on the points and under the nails of his fingers, which peels off without discharge. A dry eruption, also, between the fingers and toes, and on the palms and soles, which detaches itself, in like manner, in the form of thickened cuticle; in some spots on the latter leaving a little rawness; a suspicious excoriation on the inside of the prepuce; appearance unhealthy. Used fumigation fourteen times. Mouth made very sore, and considerable salivation. Cured.

*Remarks.*—This Portuguese being of irregular habits, it is probable that the appearances described were the sequelæ of a prior and more confirmed pox than the primary symptoms alluded to; of which he was, in all probability, as perfectly cured as he could be, in a diseased habit. The mouth became so sore, that the head was latterly excluded during the progress of fumigation.

August.—A European. A general, vesicular, watery eruption over the body, itchy, and disturbing rest; a swelling in the groin, of long standing, and glandular swelling on the right side of the neck; phymosis, and discharge; general health considerably affected; appetite bad; sleepless; spirits greatly depressed. Has used mercury at various times, for suspected venereal, in the course of the last two years. The eruption first appeared between two and three months ago, for which ointment and washes of various kinds were applied. Latterly has been taking corrosive sublimate, and using mercurial frictions, with partial good effect on the eruption, but evidently with injury to the constitution. Fumigated fourteen times. Mouth sore, and salivated. Cured.

*Remarks.*—I fancy so obstinate a complication of symptoms cannot be assigned to any other cause than a latent venereal taint. A proof, perhaps, of the system, as well as the mouth, being early affected, or at least favourably influenced by the mercury, was the healthy sleep which followed four fumigations. This amendment, however, was disturbed by a little gastric derangement, which an aperient immediately rectified. The profuse sweating, which in every instance has attended the process for a shorter or longer period, in this case soon abated; and a slight irritation of the eyes, from the vapour, was the only inconvenience afterwards. The little phymosis remaining seems to be natural. The sore mouth occasioned some suffering; but the feelings of rude health and good spirits now enjoyed, are gratefully valued by this patient.

August.—A poor patient. Considerable inflammation, with swelling and slight suppuration, above the ossa nasi; two small ulcerated openings between the cheek and right ala, discharging matter and maggots; a purulent discharge from the nostrils; œdema of the lower eyelids, closing both eyes; shooting pains of the cranium. Was fumigated fourteen times. Mouth very sore, and severely salivated.

Dismissed, cured, 1st October.

*Remarks.*—It is not easy to imagine a more loathsome object than this poor old man presented, and, although no distinct history could be obtained, it may be denominated (I presume) a form of secondary syphilis. In so virulent a degree of inflammation, threatening such destruction, but little can be conceded to fomentations, which were conjoined in the treatment, or the few grains of camphor introduced into the ulcerated openings. The symptoms began to subside speedily, as the system very early became affected,—judging from the mouth, as well as the amendment. The mouth I thought it prudent, for the sake of the neighbouring parts, to expose to the vapour, throughout the whole period of the fumigation, thereby inducing a more than usually profuse salivation, and rather severe ulceration of the mouth. The only other inconvenience experienced was a little gastric derangement, which an emetic and gentle aperient removed. A small cicatrix is the only exterior vestige of disease now remaining. The features are again natural; and a slight purulent discharge from the nostrils, which remained for some time, was cured by a gently astringent injection.

September.—Blucgwatta, shop cooly. For the last ten months or so has been in delicate health, and suffering from general pains. Complaints originated in jaundice, for which he took mercury. Is at present thin and emaciated, and general health is greatly impaired. Has used medicines of various kinds at different times. Fumigated, three times daily, with the simple oxyde of mercury, for fourteen times. Mouth tender on the second day, when the pains began to give way. Cured.

*Remarks.*—This case of chronic rheumatism, besides the speedy relief procured, is only remarkable for the fumigatory employed,—the common blue-pill mass of our Pharmacopœia.

Dhondoo Parab, Sepoy, admitted on the 3d instant. Has concealed a venereal infection for fifteen days. Penis greatly swollen, tense and very painful, with gangrenous inflammation of the prepuce, which at one place is ulcerated through, and discharging a foul, bloody pus. A poultice was applied for two days; but the progress of disease became so threatening to the penis, that fumigation was resorted to on the 5th. On the 6th, the mouth was tender, and the line of separation between the diseased and sound parts of the prepuce forming ; and, on the 7th, the prepuce dropped off, leaving a clean sore, as if circumcised, and exposing a deep foul ulceration in the body of the glans. Mouth very sore, and salivation profuse, partly from the native composition, and partly from the oxyde of mercury. Sore healed under simple dressing, and blue-stone occasionally.

*Remarks.*—This case affords an instructive lesson of the good effects of speedily impregnating the system, in cases of virulent primary venereal sore; and the simplicity of the process by which this can be accomplished, and disease arrested. Fumigation may here be said to combine all the advantages derived from the general and topical application of mercury. This patient was returned to his duty early in October, with no more mutilation than circumcision causes, although, on entering the hospital, the prognosis was so unfavourable. I have never seen circumcision by the knife heal more speedily ; a fair test, it may be allowed, of all morbid taint in the system being destroyed.

Observations.—Having professed obligations to the native practice of mercurial fumigation, and adduced examples in support of its sanative efficacy, I shall now mention the composition which is in use in this corner of India, either for adoption in all its crudeness, or to benefit by the improvement of the more cultivated judgment and systematic experience of others.

Quicksilver ʒiij. = 1 iola.

Litharge *(moordarsing,)* ʒj. ℈ij. = ½ iola.

Red lead *(sendoor,)* ʒiij.

Sulphate of copper ʒss.

The whole is well triturated together, for about two hours, with a quantity of the leaves of a jungle plant *(shetur)*, till formed into a mass, which is divided into fourteen portions, each the size of a small nutmeg, for use. One of these being laid on a small bit of tile, this is placed on live cow-dung, in an earthen dish, on the ground, between the patient's legs, who, in a sitting posture, is enveloped in a blanket. In about half an hour, the operation is over, the mercury being volatilised; and it is usual to employ this morning and evening.

The merits of such a compound must, I presume, be allowed to rest on its remedial virtues, and the nicety of analytical research forborne, in tenderness to its curative effects. But, although refinement may smile at its rudeness, and chemistry might detect much that is unscientific in a combination which such ingredients form, and perhaps caution, *a priori*, might shrink from its employment, it may be that the most fastidious will discover more that is extraneous than noxious in it. The following sentiments of Dr. Paris, in his "Pharmacologia," will be received as respectable support, at least of the latter part of this opinion. "Certain bodies," says Paris, "appear likewise to be incompatible with the compounds of lead, not from the chemical changes they induce, but from the contrary effects they produce upon the body: thus, mercury appears to invalidate their powers, and to counteract their effects, as we may have observed in treating saturnine cholic."

But, far from advocating a remedy on any ground which sound principle and experiment do not support, and (in common, probably, with most medical men,) rather inclined to be sceptical and wary in any deviation from established truths, than become the partisan of equivocal hypothesis, it will be observed, in my latter cases, that a simple oxyde of mercury was employed. It was partly the dread of the saturnine portion of the composition which made me resort to this change; but whether, in cases of phagedena, or virulent venereal ulceration, requiring speedy arresting means, lead may not be a very essential fumigating principle, must be left to experiment to decide. As the simplest form, and one always to be procured, I employed the common blue-pill mass, in the quantity of half a drachm each time, which probably, both in quality and quantity, is not far removed from the native preparation of mercury.

It is not my intention, in this communication, to enter on the merits or demerits of a plan of using mercury, which (not more favoured than others) has at various times had its abettors and detractors: neither shall I further enlarge on its modified combination. The opportunity has been afforded me of proving it favourably, and the results of my success I have confidence in giving publicity to. I have also had occasion to know that, in respectable hands elsewhere, since my first cases were promulgated, it has generally fulfilled expectations; and, from this union of success, it has been favoured with the recommendation, to the gentlemen in his division, of one of our most experienced medical officers, who superintended my hospital. In venereal affections of every stage, it is in universal use among the native population, who (so far as I have observed, and can learn,) exhibit no deforming traces, either of its baneful consequences or inertness.

Therefore, from a certain satisfactory experience, and an anticipation of the valuable purposes, in morbid conditions of the system, to which so active an agent as mercurial fumigation, may be safely applied, I hope that this little practical sketch may be acceptable.

### APPENDIX.

### *Extract of a Letter to* John Milne, Esq. M.D. Superintending Surgeon, *Konkan Division of the Army.*

As every mean which can conduce to the melioration of human suffering has its intrinsic value, and, in proportion to its success, is equally estimable, whether the fruit of philosophical research, or whether derived from the simple experience of the unscientific native of the wilderness, I shall anticipate my monthly Return, by sending a statement of the cases which have been subjected to mercurial fumigation.

It would be premature, and might seem too imaginative, perhaps, to deduce from so limited a sphere of experience the manifold advantages which time may yet demonstrate will accrue, from the introduction into our hospitals of a practice, which I am not aware has met with any attention in this country from scientific men. But, as the cases treated under my inspection were also under your superintendance, you are therefore well acquainted with their obstinacy and inveteracy, and will think (I do not doubt) that they afford ample grounds for encouraging the profession to the like practice, at least in similar cases of disease; and may, therefore, be induced to bring the same to the notice of the Medical Board for this purpose.

It would betray unpardonable ignorance in any one to claim merit for suggesting a practice in any stage of the venereal disease, which the earliest physicians employed on the first appearance of that scourge in Europe, and which in later times has been recommended to the profession by M. Lalanette in France, and Abernethy in England. This is far from my ambition: nor, indeed, would the cases I have brought forward support any such vain pretension. But in anomalous diseases, whether considered the sequelæ of syphilis, or denominated mercurial or rheumatic, (examples of which mine may be called, and most native as well as European hospitals furnish annually a longer or shorter list, in their chronic state,) there is, for the present, a novelty in the practice to regular practitioners, although, probably, as ancient as the laws of Menu to the Vytians of Hindostan.

In contemplating the extension of advantages from the employment of mercury in the form of fumigation, one dwells on the frequent, perplexing, and often deplorable, instances, in which all our hopes rest on the antidotal influence of this mineral, if it could be but brought to act on the system; and the disheartening failure in this anxious endeavour, which every practitioner has at times to lament. These few testimonials, therefore, of the almost sudden influence on the constitution of a medicine so potent in all tropical diseases, will not be deemed unworthy of some consideration, but also of more enlarged trial, not only in parallel cases, but probably in many others, which cannot fail to suggest themselves in the hour of difficulty to the reflecting mind. In the appalling suspension of the *vis vitæ* in cholera, for example, combined with the judicious administration of internal stimuli, it is surely deserving of a trial, where so many measures have failed. We are told that the ancients mixed incense, myrrh, *musk,* and other odoriferous and stimulating drugs, with their mercurial fumigatories. Might not these be advantageously combined with the mercurial fumigatory, in cholera? Indeed, there are, perhaps, none of the Indian diseases, in which instances do not at times occur, that far more desperate measures would not, even in humanity, be resorted to, were any such available. But fumigation is so easy, and so little fatiguing in its application, that, if it were but successful wherein I have presumed to suggest, it would, indeed, be an unspeakable blessing, and by no means entitled to so forbidding an epithet.

The objections which have shaken confidence in, and restricted the employment of, mercurial fumigation as a specific in lues, I would totally discard, in bringing it to notice in the treatment of diseases of climate: if the permanency of its effects are in the former questionable, its speedy influence on the system is nevertheless confessed by all its detractors. But even that opposition is losing ground, as the following extract from the best and most modern practical work extant on the Venereal Disease exemplifies:—" Mais les fumigations méritent d'être conservées en ce qu'elles sont un excellent moyen topique dans les symptomes extérieurs opiniâtres: nous en avons vu des effets surprenans et *presque subits*, principalement dans les ulcérations du fond de la gorge, dont la marche rapide nous faisait craindre la carie des vertèbres cervicales correspondantes. Nous croyons, en conséquence, que les fumigations prudemment administrées seront toujours d'un grand secours dans ces sortes de chancres vénériens, ainsi que dans ceux qui affectant le palais, le nez, la muqueuse des fosses nasales, ou des sinus frontaux, peuvent menacer d'une prompte destruction les os voisins."

Having seen some good from a practice, which has met with great neglect from the regular practitioners of this country, I have deemed it a duty to solicit your bringing the same to the notice of the Medical Board. It has also occurred to me to offer some suggestions, deduced from a few practical data, and the almost universal powers of mercury on disease, and, thus generalising, thereby incurring stricture for favouring empiricism, rather than not present so imperfect a contribution. But it is in the hope that the experience of others, or my own, will hereafter substantiate them on a basis as rational and scientific, at least, as that on which the fame of many of our best remedies rests.

*Camp, near Leverndroog; August 21st, 1824.*

